# Health, lifestyle and employment beyond state-pension age

**DOI:** 10.1186/s12889-017-4957-5

**Published:** 2017-12-20

**Authors:** Evangelia Demou, Abita Bhaskar, Taoye Xu, Daniel F. Mackay, Kate Hunt

**Affiliations:** 1 MRC/CSO Social and Public Health Sciences Unit, University of Glasgow, 200 Renfield Street, Glasgow, G2 3QB UK; 2 Public Health, Institute of Health and Wellbeing, College of Medical, Veterinary and Life Sciences, University of Glasgow, Glasgow, UK

## Abstract

**Background:**

The factors influencing one’s choice to retire vary, with financial and health considerations being some of the main factors impacting or associated with people’s timing of retirement. The aim of the study is to investigate the differences in current health and health-related behaviours, such as smoking, drinking and exercising, between people who kept on working beyond state-pension age and those who retired before or at state-pension age.

**Methods:**

Data from six waves (2003, 2008–2012) of the Scottish Health Survey (SHeS) are used. Descriptive analyses were used to characterise the population. Multivariate logistic regression was undertaken to analyse the relationship between retirement groups and gender, age, deprivation, marital status, housing tenure, general health, longstanding illness, cigarette smoking status, amount of exercise and mental health, using Stata.

**Results:**

Reporting poor self-rated health or having a long-standing illness was associated with increased odds of retiring before state pension age (SPA) in groups with a medium deprivation profile in almost all the survey years. For the least deprived there was little evidence of an association between poor health and extended-working-life, while significant associations were observed for the most deprived. An increasing trend was observed for both genders in the number of people extending their working life. Similar associations between reporting poorer self-rated health and extended working lives were observed for men and women. Distinct gender differences were observed for the associations with reporting poor mental health and no exercise. In the adjusted models, both were significantly associated with retiring at or before SPA in almost every year for women, whereas no significant associations were observed (except in 1 year) for men.

**Conclusions:**

This study shows an increasing trend in the number of people extending their working lives and demonstrates significant associations between health and lifestyle behaviours and employment status past SPA. The results suggest that good health – both physically and mentally – along with either a need or a want to stay in employment could be important reasons for continuing to work beyond SPA.

**Electronic supplementary material:**

The online version of this article (10.1186/s12889-017-4957-5) contains supplementary material, which is available to authorized users.

## Background

The definition and concept of retirement in the literature varies and usually refers to retirement as leaving paid employment [1, 2]. Denton and Spencer (2009) systematically reviewed a number of definitions of retirement and grouped them into non-participation or reduced participation in the labour force, receiving state pension, end-of-career employment, and self-assessed retirement [1]. At the same time, retirement is often described as a permanent state and an individual and voluntary choice [3].

With life expectancy rising, the UK pension system will be under pressure as more people need pensions and the pension amounts required will also increase [4–7]. Half a century ago, working life in the UK was much longer than present working lives [5]. The rate of men staying in paid employment among 55–59 year-olds dropped from 90% to 70% during the 1968 to 1990 period; for 60–64 year-olds it decreased from 80% to 50%, and for 65–69 year-olds it decreased from 30% to 15% [5]. The culmination of these issues resulted in the abolition of the mandatory retirement age in the UK and people are now encouraged to postpone their retirement [8]. The UK has been moving towards equalization of state pension age (SPA) for men and women. In 2006 the state pension age was 61.75 and 65 years for females and males, respectively [9]. According to the 2011 UK Government State Pension Age (SPA) Timetables, women’s SPA is now increasing rapidly to 65 [6]. From the end of 2018, SPA for both males and females will keep increasing until it reaches 66 by the end of 2020 and 67 during the 2026 to 2028 period [10]. The SPA for people born after the 5th of April 1969 and before the 6th of April 1977 has already reached 67 [9]. For many, state pension may be their main financial resource [11]. However, the state pension plus individuals’ previous savings (including occupational and private pensions) are often less than the amount that allow for a comfortable living [12]. Ensuring that all retired people have a ‘wealthy retirement life’ under the current pension system and policies is a major concern [8].

The factors influencing one’s choice to retire vary, with financial [13, 14], health [14–17], and education [18], being some of the main factors impacting or associated with people’s timing of retirement. Some studies investigating factors that impact on retirement have directly focused on lifestyle and behaviours (e.g. physical activity, smoking, and drinking) [19–21], while others confirm that smoking and drinking can also indirectly influence retirement timing [19–21] through the influence these behaviours have on health and/or economic status [22, 23]. A large occupational cohort study displayed evidence of an association of smoking with rising disability retirement [24]. There is also evidence that drinking habits can trigger occupational disability [25]. A study exploring employment in seafaring occupations, for instance, showed that alcohol abuse can result in diseases which may ultimately lead to early retirement [26].

Studies investigating differences in unhealthy behaviours or lifestyle choices between those who keep on working *beyond* “normal” retirement age and those who retire *before* or *at* “normal” retirement age are scarce. Previous evidence suggests that people who retire early and people who retire late tend to have different health behaviours [27], but these differences are not always quantified.

The aim of this study is to analyse the 2003 and 2008–2012 waves of the Scottish Health Survey (SHeS) and investigate, describe and understand differences in current health and lifestyle status, and how these vary between people who remain in paid employment beyond SPA and those who retire at or before SPA in Scotland.

## Methods

The Scottish Health Survey (SHeS) is carried out on behalf of the Scottish Government Health Directorates and NHS Health Scotland. It collects information on general health, investigates risk factors and explores the prevalence of particular health conditions on a representative sample of the Scottish Population [28]. The first SHeS was conducted in 1995, and collected information on people aged 16–64. It was not until the third wave (2003) that participant age limitations were lifted [9].

The SHeS is a cross-sectional survey that collects data on a wide range of health and lifestyle indicators, including self-assessed health, mental health problems, smoking, drinking and amount of physical activity carried out. This study used six waves of the SHeS to examine the relationship between economic activity and various lifestyle and health-related outcomes.

### Study population

The 2003 to 2012 SHeS waves were used. Previous SHeS waves could not be used due to lack of information on income and age when last in a paid job, and the exclusion of participants over 65 years old.

The survey waves were examined individually as it was not possible to combine data from 2008 to 11 with 2003 or 2012 as the survey waves have different designs and are weighted differently [29]. We were also interested to see if there were year by year differences, particularly around the change in state pension age in 2010.

### Health and lifestyle variables

The health and lifestyle variables examined in this study were self-rated health, long-standing illness, alcohol consumption, objectively measured body mass index (BMI), smoking status, self-reported exercise and mental health.

Self-rated health was dichotomised as good (‘very good’ and ‘good’) versus bad (‘fair’, ‘bad’ and ‘very bad’). Having a long-standing illness was coded as either yes or no. Alcohol consumption was divided into those who reported consuming up to 14 units per week (i.e. less than and including the recommended weekly limit guidelines [30]) and those who reported more than 14 units per week (i.e. those who were over the weekly limit). BMI was recoded as ‘not overweight’ (BMI less than 25 kg/m^2^) or ‘overweight’ (BMI equal to or greater than 25). Smoking status was defined as being a non-smoker (never smoker and ex-smoker) or a current smoker. Exercise was recoded as doing some exercise versus no exercise at all. Mental health was assessed using the short form of the General Health Questionnaire (GHQ-12). Respondents with GHQ scores less than 4 were said to be in ‘good’ mental health, while those with scores of 4 or more were defined as being in ‘poor’ mental health [28].

### Economic activity

As retirement age for individuals depends on the year, and in some cases also the day, they were born, the UK Government state pension age (SPA) timetable and calculator were used to estimate individual SPAs [9, 31]. In the UK, SPA for men born after 1960 is 67 years old, SPA for men born between 1955 and 1960 is 66 years old, and for men born before 1955 is 65 years old. All male respondents in our sample were required to work until 65 before reaching SPA.

For women born between 1951 and 1953 SPA differs by month and even date of birth. Therefore, SPA for women born in these years cannot be calculated with the existing available SHeS variables, as date of birth is not provided. In order to create our explanatory variable, it was necessary to request extra information from SHeS. The SHeS created two new flag variables to determine (i) whether or not a woman had reached SPA; and (ii) whether the women had worked beyond SPA or retired at or before SPA. These flag variables were provided to the research team without revealing any personal information. In the 2003–2010 waves, all female respondents were of an age where they would reach SPA at 60 and SPA could be estimated. However in the 2011 and 2012 waves, the above flag variables provided by SHeS were used to determine whether or not women had reached SPA.

After receiving the additional variables, respondents were divided into five groups based on their current economic activity, their current age and when they last had a paid job. For women, the flag variables were also used. Across all study years these groups were defined as: (1) *‘Currently working beyond SPA’* (N: M = 403, F = 812), (2) *‘Currently retired and beyond SPA: worked beyond SPA’* (N: M = 497, F = 1614), (3) *‘Retired and currently below SPA’* (N: M = 415, F = 149), (4) *‘Currently retired and beyond SPA: retired at or before SPA’* (N: M = 3965, F = 4615), (5) *‘Currently retired and beyond SPA: don’t know when they retired’* (N: M = 24, F = 186).

**Fig. 1:**
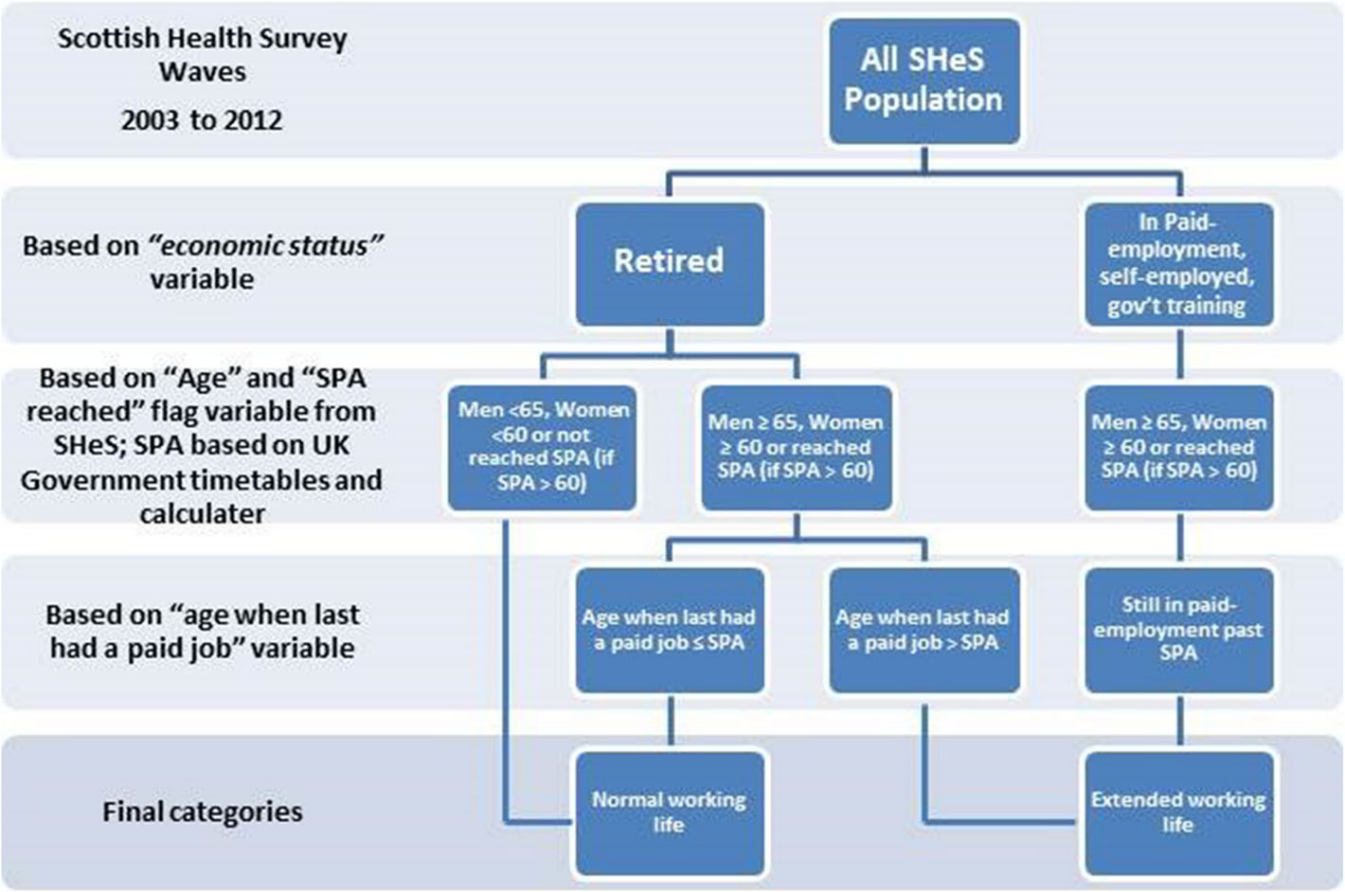
Extraction stages of SHeS study population used in this study

These groups were then amalgamated into two - those who worked beyond SPA (‘*Extended Work Life’* group – N: M = 900, F = 2426), and those who retired before/at SPA (‘*Normal Work Life’* group – N: M = 4380, F = 4764) (Fig. 1). Respondents whose retirement age was unknown were removed from the sample. Figure 1 depicts the process of extracting the study population of each group.

### Analyses

Stata/IC version 13.1 was used for the statistical analyses. Analyses were first carried out by deprivation quintile and then separately for men and women. For deprivation, the Scottish Index of Multiple Deprivation (SIMD) was used. SIMD uses a number of different area-based indicators such as income, employment, education and health to create a relative measure of deprivation for residential addresses in Scotland. For each year, associations between economic activity and the health variables were investigated using chi-square tests. Logistic regression analysis was carried out to assess the dependence of the health and lifestyle variables on employment status by deprivation category, first unadjusted, then adjusted for age, marital status, equivalised income and housing tenure.

## Results

In each wave - except for 2012 - there were approximately 900 men (min 849, max 969) and 1200 women (min 1090, max 1348). The sample size in 2012, however, was 25% smaller (661 men and 903 women). This was due to the overall SHeS survey sample being much smaller that year (4815 compared with 7544 in 2011). Table 1 presents the demographic characteristics of the sample, including gender, age, marital status and deprivation quintile.

**Table 1:** Sample demographics^a^

	Retired before/at SPA		Worked beyond SPA	
	N	%	N	%
Year				
2003	1592	17.41	549	16.51
2008	1470	16.08	469	14.10
2009	1645	17.99	598	17.98
2010	1662	18.18	598	17.98
2011	1691	18.49	632	19.00
2012	1084	11.85	480	14.43
Gender				
Male	4380	47.90	900	27.06
Female	4764	52.10	2426	72.94
	(26, 101)		(60, 103)	
Age range^b^				
45–54	78	0.85	0	0.00
55–64	1768	19.34	680	20.44
65–74	4146	45.34	1570	47.20
75+	3149	34.44	1076	32.35
Marital Status				
Single	631	6.90	126	3.79
Married & living with spouse/partner	5424	59.33	1991	59.86
Married and separated	182	1.99	58	1.74
Divorced	645	7.06	265	7.97
Widowed	2260	24.72	886	26.64
SIMD quintiles				
5 (least deprived)	1764	19.29	593	17.83
4	1970	21.54	844	25.38
3	1994	21.81	841	25.29
2	1801	19.70	608	18.28
1 (most deprived)	1615	17.66	440	13.23
Housing Tenure				
Buying a house with mortgage	781	8.62	359	10.89
Own a house outright	5815	64.17	2222	67.42
Part rent/part mortgage	54	0.60	18	0.55
Rent	2302	25.40	633	19.21
Rent free	110	1.21	64	1.94

^a^Population size per year and total population demographics

^b^participants under 45 years of age excluded

**Fig. 2:**
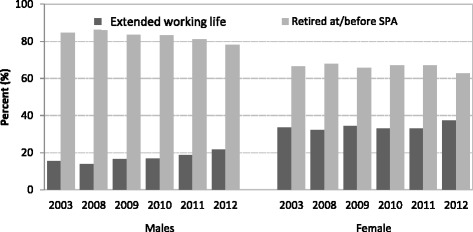
Economic activity by SHeS wave

Looking at the extended working life group by gender and year (Fig. 2), the percentage of men who worked beyond SPA in 2003 was 15.5%. This dipped slightly in 2008 (13.9%) but then increased steadily from 2009 (16.5%) until 2012 (21.8%) (Fig. 2). Among women, approximately 33.0% worked past SPA each year except 2012 when it increased to 37.0% (Fig. 2).

### Deprivation analysis

Regressions were first carried out by deprivation quintile before and after adjusting for age, gender, marital status, equivalised income and housing tenure.

#### Self-rated health

The unadjusted models (Table 2) demonstrate for the least deprived (quintile 5), poor self-rated health was significantly associated with retiring at or before SPA in two of the six survey years (2009 and 2011). However, after adjusting for confounders, this relationship was no longer seen in any of the 6 years. In quintile 4, poor self-rated health was associated with early retirement in four of the 6 years (2003, 2009–11), but significant associations were seen for 3 years (2003, 2009, 2010) in the adjusted models. In quintiles 3 and 2, a significant association between poor self-rated health and retiring at or before SPA was seen in both unadjusted and adjusted models in all six survey years, except for 2009 in the adjusted models. Among the most deprived (quintile 1) reporting poor self-rated health was associated with early retirement in three of the survey years (2009–11). These associations remained significant after adjusting for confounders (Table 2).

**Table 2:** Odds ratios and confidence intervals for poor self-rated health by economic activity (SIMD quintiles)

	5 (least deprived)		4		3		2		1 (most deprived)	
	Unadjusted OR	Adjusted OR	Unadjusted OR	Adjusted OR	Unadjusted OR	Adjusted OR	Unadjusted OR	Adjusted OR	Unadjusted OR	Adjusted OR
Poor self-rated health										
2003										
Extended Working Life	1	1	1	1	1	1	1	1	1	1
Normal Working Life	1.41 (0.83, 2.37)	1.48 (0.79, 2.77)	1.59* **(1.04, 2.42)**	2.10** **(1.26, 3.49)**	1.82** **(1.19, 2.79)**	1.89* **(1.13, 3.18)**	1.69* **(1.07, 2.66)**	1.85* **(1.08, 3.15)**	1.69 (0.94, 3.05)	1.97 (0.96, 4.06)
2008										
Extended Working Life	1	1	1	1	1	1	1	1	1	1
Normal Working Life	1.44 (0.77, 2.72)	1.83 (0.84, 3.97)	1.51 (0.96, 2.38)	1.58 (0.91, 2.72)	2.39*** **(1.46, 3.91)**	2.12* **(1.17, 3.85)**	1.72* **(1.05, 2.81)**	2.09* **(1.15, 3.80)**	1.43 (0.82, 2.49)	1.82 (0.92, 3.58)
2009										
Extended Working Life	1	1	1	1	1	1	1	1	1	1
Normal Working Life	2.11* **(1.18, 3.77)**	1.52 (0.76, 3.04)	2.57*** **(1.57, 4.20)**	2.79*** **(1.53, 5.08)**	1.69* **(1.13, 2.51)**	1.61 (0.99, 2.62)	1.72* **(1.07, 2.78)**	1.46 (0.82, 2.59)	3.43*** **(2.02, 5.82)**	3.35*** **(1.74, 6.46)**
2010										
Extended Working Life	1	1	1	1	1	1	1	1	1	1
Normal Working Life	1.33 (0.82, 2.16)	0.88 (0.49, 1.59)	2.22*** **(1.36, 3.62)**	3.07*** **(1.56, 6.04)**	2.23*** **(1.42, 3.50)**	2.57*** **(1.46, 4.51)**	2.27*** **(1.46, 3.54)**	2.25** **(1.32, 3.84)**	3.21*** **(1.95, 5.27)**	3.45*** **(1.84, 6.46)**
2011										
Extended Working Life	1	1	1	1	1	1	1	1	1	1
Normal Working Life	1.77* **(1.01, 3.11)**	1.88 (0.96, 3.67)	1.74** **(1.15, 2.62)**	1.48 (0.91, 2.42)	2.26*** **(1.53, 3.33)**	1.72* **(1.08, 2.73)**	2.12*** **(1.34, 3.37)**	2.10** **(1.19, 3.73)**	2.54*** **(1.53, 4.20)**	2.64** **(1.35, 5.14)**
2012										
Extended Working Life	1	1	1	1	1	1	1	1	1	1
Normal Working Life	0.92 (0.55, 1.56)	1.26 (0.65, 2.45)	1.26 (0.80, 2.01)	1.23 (0.72, 2.10)	2.30*** **(1.46, 3.63)**	2.73*** **(1.55, 4.82)**	2.23** **(1.29, 3.84)**	2.07* **(1.07, 4.00)**	1.11 (0.54, 2.30)	1.16 (0.47, 2.82)

*p ≤ 0.05; **p ≤ 0.01; ***p ≤ 0.001

#### Long-standing illness

In quintile 5 (least deprived), reporting a long standing illness increased the odds of retiring early only in 2010 and this significant relationship remained after adjusting for confounders. Among respondents in quintile 4, having a long-standing illness was associated with early retirement in four survey years (2003, 2009–11) in the unadjusted models. In the adjusted models, this association is no longer significant in 2010, but is also observed in 2012. In quintile 3, respondents in 2003, 2009 and 2011 who reported a long-standing illness were more likely to have retired at or before SPA. After adjustment, this relationship is also significant in 2008. In quintile 2 respondents who reported a long-standing illness in survey waves 2003, 2008–10 were significantly more likely to have retired at or before SPA, both before and after adjusting for confounders. Finally, among the most deprived (quintile 1), reporting a long-standing illness was significantly associated with retiring at or before SPA in three survey waves (2009–2011) in the unadjusted models, but only in two survey waves (2010, 2011) after adjustment for confounders (Table 3). There was no significant relationship between having a long-standing illness and normal working life (i.e. retiring at or before SPA), in any of the quintiles, in the most recent survey examined (2012) in the adjusted models.

**Table 3:** Odds ratios and confidence intervals for longstanding illness by economic activity (SIMD quintiles)

	5 (least deprived)		4		3		2		1 (most deprived)	
	Unadjusted OR	Adjusted OR	Unadjusted OR	Adjusted OR	Unadjusted OR	Adjusted OR	Unadjusted OR	Adjusted OR	Unadjusted OR	Adjusted OR
Longstanding illness										
2003										
Extended Working Life	1	1	1	1	1	1	1	1	1	1
Normal Working Life	1.24 (0.70, 2.17)	1.23 (0.64, 2.39)	1.92** **(1.25, 2.96)**	2.17** **(1.30, 3.63)**	1.71** **(1.15, 2.55)**	2.45*** **(1.49, 4.03)**	1.76** **(1.16, 2.66)**	2.06** **(1.26, 3.36)**	1.55 (0.95, 2.52)	1.32 (0.74, 2.36)
2008										
Extended Working Life	1	1	1	1	1	1	1	1	1	1
Normal Working Life	1.58 (0.94, 2.66)	1.71 (0.91, 3.19)	1.20 (0.81, 1.78)	1.25 (0.78, 2.00)	1.52 (0.98, 2.37)	1.98* **(1.14, 3.45)**	2.51*** **(1.53, 4.10)**	2.64*** **(1.47, 4.72)**	1.35 (0.73, 2.52)	1.46 (0.68, 3.16)
2009										
Extended Working Life	1	1	1	1	1	1	1	1	1	1
Normal Working Life	1.17 (0.77, 1.78)	1.02 (0.61, 1.70)	1.64* **(1.12, 2.41)**	1.70* **(1.06, 2.73)**	2.02*** **(1.38, 2.98)**	1.95** **(1.25, 3.06)**	2.05** **(1.26, 3.33)**	2.00* **(1.09, 3.67)**	2.05** **(1.23, 3.41)**	1.87 (1.00, 3.51)
2010										
Extended Working Life	1	1	1	1	1	1	1	1	1	1
Normal Working Life	2.47*** **(1.62, 3.78)**	2.27*** **(1.38, 3.72)**	1.76** **(1.18, 2.63)**	1.58 (0.97, 2.58)	1.22 (0.79, 1.87)	1.24 (0.75, 2.05)	2.55*** **(1.63, 3.99)**	2.41*** **(1.41, 4.10)**	3.01*** **(1.85, 4.91)**	2.62** **(1.42, 4.84)**
2011										
Extended Working Life	1	1	1	1	1	1	1	1	1	1
Normal Working Life	1.15 (0.74, 1.79)	1.24 (0.74, 2.08)	1.60* **(1.10, 2.34)**	1.60* **(1.01, 2.55)**	1.69** **(1.15, 2.46)**	1.62* **(1.03, 2.55)**	1.18 (0.74, 1.88)	1.52 (0.67, 2.68)	3.04*** **(1.80, 5.14)**	3.54*** **(1.85, 6.80)**
2012										
Extended Working Life	1	1	1	1	1	1	1	1	1	1
Normal Working Life	1.28 (0.78, 2.09)	1.80 (0.96, 3.38)	1.52 (0.96, 2.39)	1.71* **(1.01, 2.89)**	1.51 (0.99, 2.33)	1.28 (0.76, 2.16)	1.17 (0.65, 2.08)	1.47 (0.70, 3.10)	1.34 (0.60, 2.95)	1.31 (0.53, 3.22)

*p ≤ 0.05; **p ≤ 0.01; ***p ≤ 0.001

#### Lifestyle

There were few significant associations seen between retiring at or before SPA and lifestyle variables, including being a current smoker, not reporting doing any exercise (mainly for the most deprived) or being in poor mental health (Additional file 1; Table S21). Cross-tabulations and chi-square statistics showed no significant association between respondents (both male and female) classified as overweight (BMI greater than 25) or reporting high alcohol consumption (reporting more than 14 units per week) and extended working life. Therefore these were not included in the regression analyses (results in Additional file 1).

### Gender analysis

Regressions were carried out separately for men and women and adjusted for age, marital status, equivalised income and housing tenure and deprivation quintile (SIMD).

#### Men

Unadjusted models show that for each survey year, men reporting poor self-rated health were significantly more likely to have retired at or before SPA (Table 4). After adjusting for confounders, this association remained significant in all but two of these years (2010 and 2012). Reporting a long standing illness also increased the odds of having retired at or before SPA in each of the survey waves except 2009. After adjustment for confounders having a long-standing illness remained significantly associated with having retired at or before SPA in all but two survey years (2009 and 2010). Being a current smoker was significantly associated with retirement at or before SPA in only two surveys years (2003, 2011) in the unadjusted analysis and in none in the adjusted models. Not reporting doing any exercise was associated with increased odds of retirement at or before SPA in the later survey years (2009, 2011, 2012) in unadjusted models. After adjusting for confounders, a relationship was only seen in 2003 and 2012. Finally poor mental health was associated with retiring at or before SPA in two survey years (2008, 2009) prior to adjustment but in none after adjustment for confounders.

**Table 4:** Odds Ratios and confidence intervals for poor health by economic activity (Men)

	2003		2008		2009		2010		2011		2012	
	Unadjusted OR	Adjusted OR	Unadjusted OR	Adjusted OR	Unadjusted OR	Adjusted OR	Unadjusted OR	Adjusted OR	Unadjusted OR	Adjusted OR	Unadjusted OR	Adjusted OR
Poor self-rated health												
Extended Working Life	**1**	**1**	**1**	**1**	**1**	**1**	**1**	1	**1**	**1**	**1**	1
Normal Working Life	1.81** **(1.24, 2.66)**	1.87** **(1.19, 2.95)**	1.86** **(1.22, 2.86)**	2.42*** **(1.41, 4.15)**	2.64*** **(1.75, 3.97)**	2.13** **(1.33, 3.42)**	1.82** **(1.25, 2.67)**	1.52 (0.97, 2.41)	1.93*** **(1.35, 2.77)**	1.61* **(1.05, 2.47)**	1.61* **(1.09, 2.37)**	1.47 (0.94, 2.29)
Longstanding Illness												
Extended Working Life	**1**	**1**	**1**	**1**	1	1	**1**	1	**1**	**1**	**1**	**1**
Normal Working Life	1.68** **(1.17, 2.39)**	1.73** **(1.15, 2.62)**	1.91*** **(1.29, 2.82)**	2.13*** **(1.34, 3.38)**	1.34 (0.95, 1.89)	1.17 (0.78, 1.77)	1.75** **(1.23, 2.49)**	1.36 (0.91, 2.04)	1.79*** **(1.29, 2.49)**	2.05*** **(1.38, 3.04)**	1.83** **(1.24, 2.69)**	1.74** **(1.12, 2.71)**
Current Smoker												
Extended Working Life	**1**	1	1	1	1	1	1	1	**1**	1	1	1
Normal Working Life	1.82* **(1.04, 3.20)**	1.80 (0.91, 3.56)	1.15 (0.66, 2.03)	0.65 (0.35, 1.23)	1.20 (0.73, 1.98)	0.94 (0.51, 1.73)	1.17 (0.71, 1.93)	0.92 (0.52, 1.63)	2.01* **(1.15, 3.53)**	1.30 (0.65, 2.62)	1.28 (0.72, 2.29)	1.11 (0.57, 2.16)
No exercise												
Extended Working Life	1	**1**	1	1	**1**	1	1	1	**1**	1	**1**	**1**
Normal Working Life	1.45 (0.99, 2.10)	1.80* **(1.14, 2.85)**	1.22 (0.81, 1.82)	1.40 (0.85, 2.33)	1.43* **(1.00, 2.04)**	1.56 (1.00, 2.44)	1.24 (0.86, 1.77)	1.13 (0.73, 1.74)	1.62** **(1.29, 2.94)**	1.46 (0.95, 2.24)	1.94** **(1.29, 2.94)**	1.99** **(1.19, 3.36)**
Poor Mental Health												
Extended Working Life	1	1	**1**	1	**1**	**1**	1	1	1	1	1	1
Normal Working Life	1.24 (0.67, 2.29)	1.02 (0.52, 1.99)	2.80* **(1.00, 7.86)**	3.43 (0.81, 14.65)	3.56* **(1.28, 9.92)**	4.77* **(1.12, 20.27)**	1.86 (0.83, 4.16)	1.38 (0.60, 3.18)	1.99 (0.93, 4.24)	1.29 (0.59, 2.85)	1.26 (0.59, 2.68)	1.00 (0.42, 2.41)

*p ≤ 0.05; **p ≤ 0.01; ***p ≤ 0.001

#### Women

Unadjusted odds ratios showed that among women, poor self-rated health was significantly associated with having retired at or before SPA in each survey year (Table 5). After adjusting for confounders, these relationships remained significant. Both unadjusted and adjusted models demonstrated a relationship between reporting a long-standing illness and having retired at or before SPA in each survey year except for 2012. Being a current smoker increased the odds of retirement at or before SPA in 2008, 2010 and 2011, however after adjusting for confounders, being a smoker was only associated with having retired at or before SPA in one survey year (2010). Significant associations were demonstrated for doing no exercise and having retired at or before SPA in four of the six surveys years (2008–11). After adjusting for confounders, women who reported doing no exercise in 2003 were also found to be more likely to have retired at or before SPA. Finally, being in poor mental health was associated with retirement at or before SPA in each survey year except 2012, and in all survey years after adjusting for confounders (except 2011) (Table 5).

**Table 5:** Odds Ratios and confidence intervals for poor health by economic activity (Women)

	2003		2008		2009		2010		2011		2012	
	Unadjusted OR	Adjusted OR	Unadjusted OR	Adjusted OR	Unadjusted OR	Adjusted OR	Unadjusted OR	Adjusted OR	Unadjusted OR	Adjusted OR	Unadjusted OR	Adjusted OR
Poor self-rated health												
Extended Working Life	1	1	1	1	1	1	1	1	1	1	1	1
Normal Working Life	1.68*** **(1.31, 2.16)**	1.72*** **(1.28, 2.31)**	1.67*** **(1.27, 2.20)**	1.70*** **(1.23, 2.36)**	2.02*** **(1.56, 2.60)**	1.86*** **(1.37, 2.53)**	2.53*** **(1.97, 3.26)**	2.68*** **(1.95, 3.67)**	2.24*** **(1.75, 2.85)**	1.94*** **(1.45, 2.59)**	1.66*** **(1.25, 2.21)**	1.62** **(1.15, 2.31)**
Longstanding Illness												
Extended Working Life	1	1	1	1	1	1	1	1	1	1	1	1
Normal Working Life	1.71*** **(1.33, 2.18)**	1.84*** **(1.39, 2.46)**	1.52** **(1.17, 1.97)**	1.50** **(1.11, 2.03)**	2.05*** **(1.61, 2.60)**	1.96*** **(1.49, 2.59)**	2.34*** **(1.84, 2.96)**	2.29*** **(1.73, 3.04)**	1.59*** **(1.25, 2.01)**	1.49** **(1.13, 1.97)**	1.24 (0.93, 1.66)	1.31 (0.94, 1.85)
Current Smoker												
Extended Working Life	1	1	1	1	1	1	1	1	1	1	1	1
Normal Working Life	1.27 (0.92, 1.74)	1.19 (0.82, 1.73)	1.55* **(1.07, 2.26)**	1.35 (0.89, 2.06)	1.10 (0.81, 1.51)	0.98 (0.68, 1.42)	1.54** **(1.12, 2.13)**	1.51* **(1.01, 2.25)**	1.54* **(1.11, 2.16)**	1.37 (0.92, 2.05)	1.25 (0.85, 1.84)	1.08 (0.68, 1.70)
No exercise												
Extended Working Life	1	1	1	1	1	1	1	1	1	1	1	1
Normal Working Life	1.23 (0.96, 1.57)	1.41* **(1.05, 1.90)**	1.43** **(1.10, 1.86)**	1.47* **(1.05, 2.04)**	1.98*** **(1.55, 2.54)**	2.05*** **(1.50, 2.79)**	2.00*** **(1.57, 2.53)**	1.69*** **(1.25, 2.29)**	1.69*** **(1.34, 2.14)**	1.68*** **(1.25, 2.25)**	1.22 (0.92, 1.61)	1.27 (0.88, 1.82)
Poor Mental Health												
Extended Working Life	1	1	1	1	1	1	1	1	1	1	1	1
Normal Working Life	2.51*** **(1.64, 3.83)**	2.95*** **(1.78, 4.88)**	1.79* **(1.14, 2.80)**	1.71* **(1.03, 2.83)**	1.85** **(1.24, 2.76)**	1.94** **(1.22, 3.07)**	1.91*** **(1.31, 2.79)**	1.64* **(1.06, 2.53)**	1.73** **(1.17, 2.56)**	1.46 (0.94, 2.28)	1.33 (0.85, 2.08)	1.81* **(1.07, 3.06)**

*p ≤ 0.05; **p ≤ 0.01; ***p ≤ 0.001

## Discussion

### Summary of findings

This study explored the differences in current health and lifestyles between individuals that extend their working lives (in paid employment) beyond state pension age and those who retire at or before state pension age (normal working life) in Scotland. We demonstrated that reporting poor self-rated health is not associated with retiring at or before SPA amongst the least deprived (association only observed in 2009). For those in the middle deprivation categories (4, 3, and 2) poor-self rated health was associated with early/‘normal’ age retirement. Respondents in the most deprived SIMD category (quintile 1) were more likely to have retired early if they reported poor self-rated health, in most survey waves (2003, 2009–2011). The relationships and strengths of the associations for self-rated health and long standing illness were attenuated after adjusting for confounders. Our results showed that lifestyle-related variables of BMI and alcohol consumption were not significantly associated with the retirement variables for either men or women.

For both males and females an increasing trend in the number of people extending their working lives was observed. However, gender differences were observed for both health and lifestyle variables in relation to extended working lives. The percentage of women with extended working lives is considerably larger than that for men. This could be due to the previously lower threshold for the state pension in women. Male respondents reporting poor self-rated health in all survey waves, and limiting long-standing illness for most survey waves (apart from 2009), were more likely to have retired before SPA than those reporting good health. Poor mental health in men was associated with increased odds of retiring before SPA in the 2009 wave only. In terms of lifestyle variables, being a current smoker did not increase the odds of retiring before SPA. In 2003, 2011 and 2012, respondents who reported that they did no exercise were more likely to retire before SPA than those who did some exercise, although we acknowledge the limitations of this measure.

Females reporting poor self-rated health had higher odds of having retired at or before SPA in all waves. In all but one wave, women reporting a long standing illness also had increased odds of having retired early compared to those who do not. The association between poor mental health and extended working lives was distinctively different in females compared to males. In females reporting poor mental health was associated with having retired at or before SPA in all but one survey wave (2011), in contrast to men where only one survey wave (2009) showed significant results. Retirement at or before SPA is more likely in current female smokers (except for 2010) and in those who did not do any exercise (except 2008 and 2012).

### Our findings in context with published literature

Our results follow the expected trend of showing associations at either end of the deprivation spectrum, with the least and most deprived population more likely to stay in employment past SPA, compared with those with more average deprivation profiles [32]. Poor self-rated health and having a long-standing illness are both known to be predictors of leaving the labour market [3, 33, 34], and our results are consistent with this. The least deprived are often more likely to stay in employment longer because they are in better health [35], are in high paying, rewarding jobs [36], and have more job control [37]. In comparison, the most deprived group often have the conflicting issues of being too sick to work versus not having a private pension and needing to work to financially support themselves and their families [3].

Our findings indicate that poor mental health is more likely to be associated with women’s timing of retirement than men’s. The relationship between poor mental health and early retirement has been documented, particularly in certain female-dominated occupations, such as teaching [38, 39] or nursing [39]. However this relationship is complex with some studies suggesting that there are no significant differences between men and women [40], while others suggest there are [41]. These contrasting results by gender among studies support the need for further analyses on the impact of mental health issues on extending working lives.

The relationship between exercise and physical and mental health is complex and well established [42–44]. A previous study using US national health and insurance data has shown a relationship between lack of exercise and early retirement in the analysed population [45]. Our study demonstrated a similar association for women only. Although no association has been found between exercise and retirement age in men, we cannot discount the potential impact of exercise on extending working lives. Physical activity is recommended to sustain and improve health, for example doing regular exercise is recommended in the NICE guidelines for improving mental health [46].

Previous studies have shown that smoking is associated with early retirement due to disability [19, 43]. Our study showed no evidence for an association between smoking and retirement at or before SPA. The ‘Smoking, Health and Social Care (Scotland) Act 2005’ which came into effect in 2006 and banned smoking in enclosed public spaces in Scotland has had a significant impact on acute coronary syndrome, respiratory symptoms and pulmonary function [47, 48], often causes of leaving the labour market early [22]. This policy change may have contributed to smoking not being associated with retirement at or before the SPA in Scotland.

Obesity has been found to be a risk factor for work ability and health-related early retirement in a number of studies [49, 50]. However our study showed no association between being overweight or obese and having retired at or before SPA. Similar to the case of exercise and health, the relationship between obesity, health and work ability is intricate and difficult to disentangle. Therefore this lack of association may be due to other reasons for leaving the paid workforce early. Robroek et al. [51] found that being obese or overweight increased the risk of leaving the labour market due to disability, while Christensen and Kallestrup-Lamb (2012) did not find a similar relationship between obesity and unemployment or early retirement [41].

Contrary to literature demonstrating increased odds of early retirement [19, 22, 25] with excess alcohol consumption, our study found no association between alcohol consumption over recommended weekly limits and having retired at or before SPA.

### Study strengths

The present study was based on the Scottish Health Survey. The use of SHeS data for secondary analysis for research purposes is free of charge from the government’s website. SHeS provides representative data for all adults in the Scottish population. Multiple survey waves allow us to examine changes over time, while the large sample size allows for adjustment for a number of confounding variables.

While there are many studies looking at health after retirement, to our knowledge this is the first study to examine the relationship between health and lifestyles and extended working lives (beyond SPA) in Scotland. As the working age population gets older [52] and policy changes are geared towards extending working lives [53], this study could help policy makers, employers and employees understand how the health and lifestyle choices of the aging working population vary by deprivation status and are associated with their ability to remain in employment or not.

### Study limitations

The Scottish Health Survey was set up for a different purpose so a number of relevant variables are not available, e.g. retirement age and reason, job family and whether or not one has a private and/or employer’s pension, and some variables (such as that for physical activity, here exercise) were rather crude. Also, limitations of the variables available means we are not able to assess whether someone does other ‘work’, as in purposeful and important activity, even though retired (e.g. carer, volunteering), which could possibly impact on health in a similar way to paid employment. Some individuals would have retired many years before the data were collected, and hence it is not possible to explore associations between lifestyles and health status at their actual retirement age. Most of the SHeS data were self-reported, so there are potential issues with reliability and recollection bias especially for some variables. Furthermore, the cross sectional nature of the survey, along with the limitation of not having relevant health and lifestyle information at the exact time of retirement, means that causal associations and the direction of such associations cannot be determined.

## Conclusions

We have shown in our study that a number of outcomes including deprivation status and health factors were associated with retirement at or before the state pension age and that there were distinct differences by gender. This suggests that good health – both physical and mental –are important factors associated with working beyond state pension age. It is widely thought that being in employment is generally good for health and overall wellbeing and therefore staying in employment longer may contribute to better health and wellbeing [54, 55]. However, supporting a workforce which includes more people beyond SPA will need workplace policies, initiatives and interventions to support both the health and employment needs of this ageing workforce and account for the gender differences observed. Understanding the health, social circumstances and lifestyles of an older workforce can inform health practitioners, employers and policy makers as well as provide the knowledge base for the development and/or tailoring of interventions to support extended working lives.

## Additional file

Additional file 1: Health, lifestyle and employment beyond state-pension age. A study using the Scottish Health Survey (SHeS). Supplementary Material. The additional file contains tables detailing the overall differences in economic activity for men (**Tables S1-S10**) and women (**Tables S11-S20**) for the study years 2003–2012 and by self-rated health, longstanding illness, BMI, mental health, smoking status, alcohol consumption, exercise level, deprivation category and equivalised income. **Table S21** presents the odds ratios for smoking, exercise and poor mental health by deprivation category (SIMD quintiles). (DOCX 46 kb)
