# Supplementary material for: Health, lifestyle and employment beyond state-pension age
Source: BMC Public Health. 2017 Dec 20;17:971. doi: 10.1186/s12889-017-4957-5 (PMC5738753; doi:10.1186/s12889-017-4957-5)
Supplement: Supplementary file 1 — Health, lifestyle and employment beyond state-pension age. A study using the Scottish Health Survey (SHeS). Supplementary Material. The additional file contains tables detailing the overall differences in economic activity for men (Tables S1-S10) and women (Tables S11-S20) for the study years 2003–2012 and by self-rated health, longstanding illness, BMI, mental health, smoking status, alcohol consumption, exercise level, deprivation category and equivalised income. Table S21 presents the odds ratios for smoking, exercise and poor mental health by deprivation category (SIMD quintiles). (DOCX 46 kb) [file 12889_2017_4957_MOESM1_ESM.docx]

**For men**

**Table S1: Differences in economic activity for men 2003-2012**

|  | 2003 | 2008 | 2009 | 2010 | 2011 | 2012 | χ^2^ | *P* |
| --- | --- | --- | --- | --- | --- | --- | --- | --- |
| Worked past SPA | 15.47 | 13.90 | 16.51 | 17.72 | 19.37 | 21.92 | *22.1* | *0.000* |
| Retired at/before SPA | 84.53 | 86.10 | 83.49 | 82.28 | 80.63 | 78.08 |  |  |

**Table S2: Cross-tabulation of Economic Activity by Self Rated Health for men over the years 2003-2012**

|  |  | Worked past SPA | Retired at/ before SPA | χ^2^ | *P* |
| --- | --- | --- | --- | --- | --- |
| 2003 | Good | 70.34 | 56.69 | 9.4 | 0.002 |
|  | Bad | 29.66 | 43.31 |  |  |
| 2008 | Good | 72.03 | 58.00 | 8.3 | 0.004 |
|  | Bad | 27.97 | 42.00 |  |  |
| 2009 | Good | 79.38 | 59.33 | 22.9 | 0.000 |
|  | Bad | 20.63 | 40.67 |  |  |
| 2010 | Good | 72.08 | 59.05 | 9.1 | 0.003 |
|  | Bad | 27.92 | 40.95 |  |  |
| 2011 | Good | 72.63 | 58.66 | 11.9 | 0.001 |
|  | Bad | 27.37 | 41.34 |  |  |
| 2012 | Good | 67.36 | 56.14 | 5.8 | 0.016 |
|  | Bad | 32.64 | 43.86 |  |  |

**Table S3: Cross-tabulation of Economic Activity by Long-Standing Illness for men over the years 2003-2012**

|  |  | Worked past SPA | Retired at/ before SPA | χ^2^ | *P* |
| --- | --- | --- | --- | --- | --- |
| 2003 | No | 47.59 | 35.15 | 8.1 | 0.004 |
|  | Yes | 52.41 | 64.85 |  |  |
| 2008 | No | 50.85 | 35.16 | 10.7 | 0.001 |
|  | Yes | 49.15 | 64.84 |  |  |
| 2009 | No | 43.75 | 36.71 | 2.8 | 0.094 |
|  | Yes | 56.25 | 63.29 |  |  |
| 2010 | No | 43.79 | 31.00 | 9.3 | 0.002 |
|  | Yes | 56.21 | 69.00 |  |  |
| 2011 | No | 45.81 | 32.48 | 11.3 | 0.001 |
|  | Yes | 54.19 | 67.52 |  |  |
| 2012 | No | 40.28 | 26.95 | 9.5 | 0.002 |
|  | Yes | 59.72 | 73.05 |  |  |

**Table S4: Cross-tabulation of Economic Activity by BMI for men over the years 2003-2012**

|  |  | Worked past SPA | Retired at/ before SPA | χ^2^ | *P* |
| --- | --- | --- | --- | --- | --- |
| 2003 | Not overweight | 21.50 | 24.88 | 0.6 | 0.451 |
|  | Overweight | 78.50 | 75.12 |  |  |
| 2008 | Not overweight | 19.42 | 18.43 | 0.1 | 0.812 |
|  | Overweight | 80.58 | 81.57 |  |  |
| 2009 | Not overweight | 20.77 | 25.04 | 1.1 | 0.300 |
|  | Overweight | 79.23 | 74.96 |  |  |
| 2010 | Not overweight | 23.02 | 22.92 | 0.0 | 0.981 |
|  | Overweight | 76.98 | 77.08 |  |  |
| 2011 | Not overweight | 19.59 | 19.51 | 0.0 | 0.982 |
|  | Overweight | 80.41 | 80.49 |  |  |
| 2012 | Not overweight | 15.45 | 20.43 | 1.5 | 0.218 |
|  | Overweight | 84.55 | 79.57 |  |  |

**Table S5: Cross-tabulation of Economic Activity by Mental Health (GHQ) for men over the years 2003-2012**

|  |  | Worked past SPA | Retired at/ before SPA | χ^2^ | *P* |
| --- | --- | --- | --- | --- | --- |
| 2003 | Good Mental Health | 89.92 | 87.81 | 0.5 | 0.494 |
|  | Poor Mental Health | 10.08 | 12.19 |  |  |
| 2008 | Good Mental Health | 96.12 | 89.83 | 4.2 | 0.041 |
|  | Poor Mental Health | 3.88 | 10.17 |  |  |
| 2009 | Good Mental Health | 97.06 | 90.26 | 6.7 | 0.010 |
|  | Poor Mental Health | 2.94 | 9.74 |  |  |
| 2010 | Good Mental Health | 94.96 | 91.44 | 1.9 | 0.164 |
|  | Poor Mental Health | 5.04 | 8.56 |  |  |
| 2011 | Good Mental Health | 94.67 | 90.21 | 3.0 | 0.085 |
|  | Poor Mental Health | 5.33 | 9.79 |  |  |
| 2012 | Good Mental Health | 93.13 | 91.69 | 0.3 | 0.592 |
|  | Poor Mental Health | 6.87 | 8.31 |  |  |

**Table S6: Cross-tabulation of Economic Activity by Smoking Status for men over the years 2003-2012**

|  |  | Worked past SPA | Retired at/ before SPA | χ^2^ | *P* |
| --- | --- | --- | --- | --- | --- |
| 2003 | Non-smoker | 89.51 | 82.43 | 4.4 | 0.036 |
|  | Smoker | 10.49 | 17.57 |  |  |
| 2008 | Non-smoker | 86.44 | 84.68 | 0.2 | 0.620 |
|  | Smoker | 13.56 | 15.32 |  |  |
| 2009 | Non-smoker | 86.71 | 84.43 | 0.5 | 0.465 |
|  | Smoker | 13.29 | 15.57 |  |  |
| 2010 | Non-smoker | 86.36 | 84.71 | 0.3 | 0.603 |
|  | Smoker | 13.64 | 15.29 |  |  |
| 2011 | Non-smoker | 91.62 | 84.14 | 6.5 | 0.011 |
|  | Smoker | 8.38 | 15.86 |  |  |
| 2012 | Non-smoker | 88.81 | 85.96 | 0.8 | 0.377 |
|  | Smoker | 11.19 | 14.04 |  |  |

**Table S7: Cross-tabulation of Economic Activity by Alcohol Consumption for men over the years 2003-2012**

|  |  | Worked past SPA | Retired at/ before SPA | χ^2^ | *P* |
| --- | --- | --- | --- | --- | --- |
| 2003 | Under limit | 71.83 | 66.96 | 1.3 | 0.253 |
|  | Over limit | 28.17 | 33.04 |  |  |
| 2008 | Under limit | 62.71 | 67.17 | 0.9 | 0.341 |
|  | Over limit | 37.29 | 32.83 |  |  |
| 2009 | Under limit | 72.78 | 68.44 | 1.7 | 0.280 |
|  | Over limit | 27.22 | 31.56 |  |  |
| 2010 | Under limit | 59.48 | 66.57 | 2.8 | 0.094 |
|  | Over limit | 40.52 | 33.43 |  |  |
| 2011 | Under limit | 69.66 | 68.37 | 0.1 | 0.739 |
|  | Over limit | 30.34 | 31.63 |  |  |
| 2012 | Under limit | 69.93 | 67.71 | 0.3 | 0.614 |
|  | Over limit | 30.07 | 32.29 |  |  |

**Table S8: Cross-tabulation of Economic Activity by Exercise levels for men over the years 2003-2012**

|  |  | Worked past SPA | Retired at/ before SPA | χ^2^ | *P* |
| --- | --- | --- | --- | --- | --- |
| 2003 | Some exercise | 66.43 | 57.79 | 3.7 | 0.053 |
|  | No exercise | 33.57 | 42.21 |  |  |
| 2008 | Some exercise | 64.41 | 59.78 | 0.9 | 0.340 |
|  | No exercise | 35.59 | 40.22 |  |  |
| 2009 | Some exercise | 64.78 | 56.20 | 4.0 | 0.045 |
|  | No exercise | 35.22 | 43.80 |  |  |
| 2010 | Some exercise | 62.99 | 59.05 | 0.8 | 0.366 |
|  | No exercise | 37.01 | 40.95 |  |  |
| 2011 | Some exercise | 67.60 | 56.85 | 6.9 | 0.009 |
|  | No exercise | 32.40 | 43.15 |  |  |
| 2012 | Some exercise | 74.13 | 60.04 | 9.5 | 0.002 |
|  | No exercise | 25.87 | 39.96 |  |  |

**Table S9: Cross-tabulation of Economic Activity by deprivation (SIMD quintiles) for men over the years 2003-2012**

|  |  | Worked past SPA | Retired at/ before SPA | χ^2^ | *P* |
| --- | --- | --- | --- | --- | --- |
| 2003 | 5: Least Deprived | 15.17 | 19.57 | 11.6 | 0.020 |
|  | 4 | 24.14 | 20.96 |  |  |
|  | 3 | 28.97 | 22.85 |  |  |
|  | 2 | 24.14 | 19.82 |  |  |
|  | 1: Most deprived | 7.59 | 16.79 |  |  |
| 2008 | 5: Least Deprived | 18.64 | 17.24 | 5.0 | 0.290 |
|  | 4 | 30.51 | 24.62 |  |  |
|  | 3 | 22.88 | 22.30 |  |  |
|  | 2 | 19.49 | 20.38 |  |  |
|  | 1: Most deprived | 8.47 | 15.46 |  |  |
| 2009 | 5: Least Deprived | 18.13 | 22.13 | 14.3 | 0.006 |
|  | 4 | 31.25 | 20.89 |  |  |
|  | 3 | 28.75 | 23.73 |  |  |
|  | 2 | 11.25 | 18.17 |  |  |
|  | 1: Most deprived | 10.63 | 15.08 |  |  |
| 2010 | 5: Least Deprived | 27.27 | 20.28 | 11.4 | 0.022 |
|  | 4 | 21.43 | 20.98 |  |  |
|  | 3 | 24.68 | 19.16 |  |  |
|  | 2 | 16.23 | 20.56 |  |  |
|  | 1: Most deprived | 10.39 | 19.02 |  |  |
| 2011 | 5: Least Deprived | 18.44 | 20.27 | 9.8 | 0.045 |
|  | 4 | 26.82 | 22.15 |  |  |
|  | 3 | 26.26 | 20.54 |  |  |
|  | 2 | 18.44 | 18.66 |  |  |
|  | 1: Most deprived | 10.06 | 18.39 |  |  |
| 2012 | 5: Least Deprived | 21.53 | 22.03 | 9.9 | 0.043 |
|  | 4 | 28.47 | 22.61 |  |  |
|  | 3 | 29.86 | 23.20 |  |  |
|  | 2 | 13.89 | 19.10 |  |  |
|  | 1: Most deprived | 6.25 | 13.06 |  |  |

**Table S10: Mann-Whitney test comparing equivalised income for men by Economic Activity**

|  | Economic Activity | Median | P |
| --- | --- | --- | --- |
| 2003 | Worked past SPA | 14300.00 | 0.986 |
|  | Retired before/at SPA | 14300.00 |  |
| 2008 | Worked past SPA | 18040.16 | 0.025 |
|  | Retired before/at SPA | 16900.00 |  |
| 2009 | Worked past SPA | 21056.34 | 0.019 |
|  | Retired before/at SPA | 16900.00 |  |
| 2010 | Worked past SPA | 22100.00 | 0.001 |
|  | Retired before/at SPA | 16900.00 |  |
| 2011 | Worked past SPA | 19500.00 | 0.017 |
|  | Retired before/at SPA | 19180.33 |  |
| 2012 | Worked past SPA | 23442.62 | 0.072 |
|  | Retired before/at SPA | 19500.00 |  |

**For women**

**Table S11: Differences in economic activity for women 2003-2012**

|  | 2003 | 2008 | 2009 | 2010 | 2011 | 2012 | χ^2^ | *P* |
| --- | --- | --- | --- | --- | --- | --- | --- | --- |
| Worked past SPA | 33.55 | 32.2 | 34.38 | 32.94 | 33.04 | 37.21 | *7.0* | *0.224* |
| Retired at/before SPA | 66.45 | 67.8 | 65.62 | 67.06 | 66.96 | 62.79 |  |  |

**Table S12: Cross-tabulation of Economic Activity by Self Rated Health for women over the years 2003-2012**

|  |  | Worked past SPA | Retired at/ before SPA | χ^2^ | *P* |
| --- | --- | --- | --- | --- | --- |
| 2003 | Good | 67.08 | 54.75 | 16.9 | 0.000 |
|  | Bad | 32.92 | 45.25 |  |  |
| 2008 | Good | 70.66 | 59.00 | 13.8 | 0.000 |
|  | Bad | 29.34 | 41.00 |  |  |
| 2009 | Good | 74.43 | 59.09 | 29.5 | 0.000 |
|  | Bad | 25.57 | 40.91 |  |  |
| 2010 | Good | 75.45 | 54.82 | 53.6 | 0.000 |
|  | Bad | 24.55 | 45.18 |  |  |
| 2011 | Good | 72.19 | 53.70 | 43.1 | 0.000 |
|  | Bad | 27.81 | 46.30 |  |  |
| 2012 | Good | 68.66 | 56.89 | 12.3 | 0.000 |
|  | Bad | 31.34 | 43.11 |  |  |

**Table S13: Cross-tabulation of Economic Activity by Long-Standing Illness for women over the years 2003-2012**

|  |  | Worked past SPA | Retired at/ before SPA | χ^2^ | *P* |
| --- | --- | --- | --- | --- | --- |
| 2003 | No | 45.79 | 33.13 | 18.4 | 0.000 |
|  | Yes | 54.21 | 66.88 |  |  |
| 2008 | No | 43.30 | 33.42 | 10.0 | 0.002 |
|  | Yes | 56.70 | 66.58 |  |  |
| 2009 | No | 48.17 | 31.22 | 35.4 | 0.000 |
|  | Yes | 51.83 | 68.78 |  |  |
| 2010 | No | 46.62 | 27.21 | 50.3 | 0.000 |
|  | Yes | 53.38 | 72.79 |  |  |
| 2011 | No | 39.29 | 28.98 | 14.7 | 0.000 |
|  | Yes | 60.71 | 71.02 |  |  |
| 2012 | No | 35.12 | 30.34 | 2.2 | 0.137 |
|  | Yes | 64.88 | 69.66 |  |  |

**Table S14: Cross-tabulation of Economic Activity by BMI for women over the years 2003-2012**

|  |  | Worked past SPA | Retired at/ before SPA | χ^2^ | *P* |
| --- | --- | --- | --- | --- | --- |
| 2003 | Not overweight | 24.26 | 26.73 | 0.6 | 0.420 |
|  | Overweight | 75.74 | 73.27 |  |  |
| 2008 | Not overweight | 26.99 | 26.85 | 0.0 | 0.964 |
|  | Overweight | 73.01 | 73.15 |  |  |
| 2009 | Not overweight | 28.94 | 28.40 | 0.0 | 0.856 |
|  | Overweight | 71.06 | 71.60 |  |  |
| 2010 | Not overweight | 23.43 | 29.33 | 4.1 | 0.044 |
|  | Overweight | 76.57 | 70.67 |  |  |
| 2011 | Not overweight | 27.90 | 29.19 | 0.2 | 0.663 |
|  | Overweight | 72.10 | 70.81 |  |  |
| 2012 | Not overweight | 28.74 | 26.61 | 0.4 | 0.539 |
|  | Overweight | 71.26 | 73.39 |  |  |

**Table S15: Cross-tabulation of Economic Activity by Mental Health (GHQ) for women over the years 2003-2012**

|  |  | Worked past SPA | Retired at/ before SPA | χ^2^ | *P* |
| --- | --- | --- | --- | --- | --- |
| 2003 | Good Mental Health | 92.20 | 82.51 | 19.0 | 0.000 |
|  | Poor Mental Health | 7.80 | 17.49 |  |  |
| 2008 | Good Mental Health | 91.32 | 85.48 | 6.5 | 0.011 |
|  | Poor Mental Health | 8.68 | 14.52 |  |  |
| 2009 | Good Mental Health | 90.93 | 84.42 | 9.3 | 0.002 |
|  | Poor Mental Health | 9.07 | 15.58 |  |  |
| 2010 | Good Mental Health | 90.25 | 82.88 | 11.6 | 0.001 |
|  | Poor Mental Health | 9.75 | 17.13 |  |  |
| 2011 | Good Mental Health | 91.11 | 85.57 | 7.6 | 0.006 |
|  | Poor Mental Health | 8.89 | 14.43 |  |  |
| 2012 | Good Mental Health | 89.26 | 86.19 | 1.6 | 0.206 |
|  | Poor Mental Health | 10.74 | 13.81 |  |  |

**Table S16: Cross-tabulation of Economic Activity by Smoking Status for women over the years 2003-2012**

|  |  | Worked past SPA | Retired at/ before SPA | χ^2^ | *P* |
| --- | --- | --- | --- | --- | --- |
| 2003 | Non-smoker | 83.87 | 80.38 | 2.2 | 0.140 |
|  | Smoker | 16.13 | 19.63 |  |  |
| 2008 | Non-smoker | 88.00 | 82.52 | 5.4 | 0.020 |
|  | Smoker | 12.00 | 17.48 |  |  |
| 2009 | Non-smoker | 84.47 | 83.13 | 0.4 | 0.539 |
|  | Smoker | 15.53 | 16.87 |  |  |
| 2010 | Non-smoker | 86.94 | 81.17 | 7.0 | 0.008 |
|  | Smoker | 13.06 | 18.83 |  |  |
| 2011 | Non-smoker | 88.30 | 83.01 | 6.6 | 0.010 |
|  | Smoker | 11.70 | 16.99 |  |  |
| 2012 | Non-smoker | 86.61 | 83.77 | 1.3 | 0.251 |
|  | Smoker | 13.39 | 16.23 |  |  |

**Table S17: Cross-tabulation of Economic Activity by Alcohol Consumption for women over the years 2003-2012**

|  |  | Worked past SPA | Retired at/ before SPA | χ^2^ | *P* |
| --- | --- | --- | --- | --- | --- |
| 2003 | Under limit | 94.29 | 92.49 | 1.4 | 0.245 |
|  | Over limit | 5.71 | 7.51 |  |  |
| 2008 | Under limit | 86.86 | 90.11 | 2.6 | 0.108 |
|  | Over limit | 13.14 | 9.89 |  |  |
| 2009 | Under limit | 89.70 | 90.31 | 0.1 | 0.730 |
|  | Over limit | 10.30 | 9.69 |  |  |
| 2010 | Under limit | 87.78 | 88.90 | 0.4 | 0.546 |
|  | Over limit | 12.22 | 11.10 |  |  |
| 2011 | Under limit | 87.20 | 88.39 | 0.4 | 0.523 |
|  | Over limit | 12.80 | 11.61 |  |  |
| 2012 | Under limit | 87.80 | 89.05 | 0.3 | 0.569 |
|  | Over limit | 12.20 | 10.95 |  |  |

**Table S18: Cross-tabulation of Economic Activity by Exercise levels for women over the years 2003-2012**

|  |  | Worked past SPA | Retired at/ before SPA | χ^2^ | *P* |
| --- | --- | --- | --- | --- | --- |
| 2003 | Some exercise | 57.86 | 52.76 | 2.8 | 0.095 |
|  | No exercise | 42.14 | 47.24 |  |  |
| 2008 | Some exercise | 65.04 | 56.58 | 7.0 | 0.008 |
|  | No exercise | 34.96 | 43.42 |  |  |
| 2009 | Some exercise | 70.71 | 54.90 | 30.0 | 0.000 |
|  | No exercise | 29.29 | 45.10 |  |  |
| 2010 | Some exercise | 67.95 | 51.50 | 32.8 | 0.000 |
|  | No exercise | 32.05 | 48.50 |  |  |
| 2011 | Some exercise | 64.82 | 52.13 | 19.8 | 0.000 |
|  | No exercise | 35.18 | 47.87 |  |  |
| 2012 | Some exercise | 62.39 | 57.67 | 1.9 | 0.163 |
|  | No exercise | 37.61 | 42.33 |  |  |

**Table S19: Cross-tabulation of Economic Activity by deprivation (SIMD quintiles) for women over the years 2003-2012**

|  |  | Worked past SPA | Retired at/ before SPA | χ^2^ | *P* |
| --- | --- | --- | --- | --- | --- |
| 2003 | 5: Least Deprived | 14.60 | 21.38 | 13.5 | 0.009 |
|  | 4 | 21.04 | 21.50 |  |  |
|  | 3 | 25.25 | 21.63 |  |  |
|  | 2 | 23.76 | 17.88 |  |  |
|  | 1: Most deprived | 15.35 | 17.63 |  |  |
| 2008 | 5: Least Deprived | 14.81 | 16.24 | 4.5 | 0.345 |
|  | 4 | 28.77 | 23.68 |  |  |
|  | 3 | 22.79 | 22.19 |  |  |
|  | 2 | 18.52 | 19.35 |  |  |
|  | 1: Most deprived | 15.10 | 18.54 |  |  |
| 2009 | 5: Least Deprived | 19.86 | 19.86 | 3.3 | 0.518 |
|  | 4 | 24.43 | 20.22 |  |  |
|  | 3 | 23.06 | 24.76 |  |  |
|  | 2 | 17.12 | 18.66 |  |  |
|  | 1: Most deprived | 15.53 | 16.51 |  |  |
| 2010 | 5: Least Deprived | 18.69 | 17.92 | 6.1 | 0.191 |
|  | 4 | 23.87 | 20.24 |  |  |
|  | 3 | 20.95 | 18.58 |  |  |
|  | 2 | 19.59 | 23.23 |  |  |
|  | 1: Most deprived | 16.89 | 20.02 |  |  |
| 2011 | 5: Least Deprived | 16.11 | 18.85 | 16.5 | 0.002 |
|  | 4 | 26.27 | 21.68 |  |  |
|  | 3 | 27.37 | 21.35 |  |  |
|  | 2 | 16.78 | 17.97 |  |  |
|  | 1: Most deprived | 13.47 | 20.15 |  |  |
| 2012 | 5: Least Deprived | 21.13 | 20.28 | 14.8 | 0.005 |
|  | 4 | 24.70 | 21.34 |  |  |
|  | 3 | 29.17 | 22.22 |  |  |
|  | 2 | 16.37 | 20.81 |  |  |
|  | 1: Most deprived | 8.63 | 15.34 |  |  |

**Table S20: Mann-Whitney test comparing equivalised income for women by Economic Activity**

|  | Economic Activity | Median | P |
| --- | --- | --- | --- |
| 2003 | Worked past SPA | 14300.00 | 0.128 |
|  | Retired before/at SPA | 11700.00 |  |
| 2008 | Worked past SPA | 14918.03 | 0.247 |
|  | Retired before/at SPA | 14918.03 |  |
| 2009 | Worked past SPA | 19180.33 | 0.049 |
|  | Retired before/at SPA | 16900.00 |  |
| 2010 | Worked past SPA | 19180.33 | 0.009 |
|  | Retired before/at SPA | 16900.00 |  |
| 2011 | Worked past SPA | 19500.00 | 0.005 |
|  | Retired before/at SPA | 17809.33 |  |
| 2012 | Worked past SPA | 19500.00 | 0.004 |
|  | Retired before/at SPA | 19180.33 |  |

**Table S21. Odds ratios for smoking, exercise and poor mental health by deprivation category (SIMD quintiles)**

|  | 5 (least) | |  | 4 | |  | 3 | |  | 2 | |  | 1 (most) | |
| --- | --- | --- | --- | --- | --- | --- | --- | --- | --- | --- | --- | --- | --- | --- |
|  | Unadjusted | Adjusted |  | Unadjusted | Adjusted |  | Unadjusted | Adjusted |  | Unadjusted | Adjusted |  | Unadjusted | Adjusted |
| Current Smoker |  |  |  |  |  |  |  |  |  |  |  |  |  |  |
| 2003 | 1.85* | 3.15** |  | 1.42 | 1.08 |  | 0.95 | 0.88 |  | 0.91 | 1.12 |  | 2.01 | 1.43 |
| 2008 | 1.40 | 0.92 |  | 1.02 | 0.86 |  | 1.30 | 1.28 |  | 1.53 | 1.20 |  | 1.66 | 1.21 |
| 2009 | 0.75 | 0.57 |  | 1.28 | 1.12 |  | 0.92 | 0.86 |  | 0.92 | 0.74 |  | 1.54 | 1.35 |
| 2010 | 0.74 | 0.94 |  | 1.66 | 1.23 |  | 0.87 | 0.91 |  | 1.27 | 0.99 |  | 2.57** | 3.42** |
| 2011 | 0.79 | 0.70 |  | 1.28 | 0.99 |  | 1.59 | 1.52 |  | 2.44* | 2.45* |  | 1.54 | 0.40 |
| 2012 | 1.87 | 2.32 |  | 1.04 | 1.11 |  | 1.29 | 1.70 |  | 0.93 | 0.76 |  | 0.86 | 0.58 |
| No exercise |  |  |  |  |  |  |  |  |  |  |  |  |  |  |
| 2003 | 1.16 | 1.00 |  | 1.40 | 1.99** |  | 1.06 | 1.05 |  | 1.12 | 1.66 |  | 1.31 | 1.84 |
| 2008 | 1.73 | 2.03 |  | 0.91 | 0.90 |  | 1.67* | 1.78 |  | 1.72* | 1.64 |  | 0.92 | 1.28 |
| 2009 | 1.43 | 1.24 |  | 1.49* | 1.72* |  | 1.82** | 1.84* |  | 2.14** | 2.02* |  | 2.55*** | 3.61*** |
| 2010 | 1.26 | 1.03 |  | 1.58* | 1.37 |  | 1.77** | 1.73* |  | 1.39 | 1.41 |  | 2.51*** | 2.48** |
| 2011 | 1.59 | 1.65 |  | 0.97 | 0.77 |  | 1.78** | 1.99** |  | 2.23*** | 2.98*** |  | 2.12** | 1.95* |
| 2012 | 0.84 | 0.79 |  | 1.63* | 2.76** |  | 1.74* | 1.81* |  | 1.22 | 1.36 |  | 0.88 | 0.81 |
| Poor mental health |  |  |  |  |  |  |  |  |  |  |  |  |  |  |
| 2003 | 1.53 | 1.96 |  | 2.65** | 3.83** |  | 2.14* | 2.15 |  | 0.83 | 1.06 |  | 4.23 | 4.00 |
| 2008 | 1.76 | 3.91 |  | 1.20 | 1.03 |  | 2.68 | 2.80 |  | 1.34 | 1.40 |  | 1.86 | 2.09 |
| 2009 | 0.79 | 1.01 |  | 1.68 | 1.87 |  | 1.71 | 2.31 |  | 1.70 | 2.52 |  | 5.72** | 9.00** |
| 2010 | 1.11 | 1.00 |  | 1.02 | 1.32 |  | 1.59 | 1.75 |  | 1.46 | 1.20 |  | 4.12** | 4.15* |
| 2011 | 1.29 | 1.58 |  | 1.92 | 1.21 |  | 1.37 | 1.16 |  | 1.32 | 1.39 |  | 2.14 | 2.27 |
| 2012 | 0.79 | 0.95 |  | 0.79 | 0.69 |  | 1.20 | 2.34 |  | 2.10 | 7.94* |  | 1.57 | 2.22 |
